# HPLC-DAD Based Polyphenolic Profiling and Evaluation of Pharmacological Attributes of *Putranjiva roxburghii* Wall.

**DOI:** 10.3390/molecules27010068

**Published:** 2021-12-23

**Authors:** Adila Nazli, Muhammad Zafar Irshad Khan, Madiha Ahmed, Nosheen Akhtar, Mohammad K. Okla, Abdulrahman Al-Hashimi, Wahidah H. Al-Qahtani, Hamada Abdelgawad, Ihsan-ul- Haq

**Affiliations:** 1 Department of Pharmacy, Faculty of Biological Sciences, Quaid-i-Azam University, Islamabad 45320, Pakistan; adilanazli44@gmail.com; 2 Chongqing Key Laboratory of Natural Product Synthesis and Drug Research, School of Pharmaceutical Sciences, Chongqing University, Chongqing 401331, China; 3 College of Pharmaceutical Sciences, Zhejiang University, Hangzhou 310058, China; m.zafarirshad@yahoo.com; 4 Shifa College of Pharmaceutical Sciences, Shifa Tameer-e-Millat University, Islamabad 44000, Pakistan; 5 Department of Biological Sciences, National University of Medical Sciences, Rawalpindi 43600, Pakistan; nosheenakhtar@numspak.edu.pk; 6 Botany and Microbiology Department, College of Science, King Saud University, Riyadh 11451, Saudi Arabia; okla103@yahoo.com (M.K.O.); al-ghamd@gmail.com (A.A.-H.); 7 Department of Food Sciences & Nutrition, College of Food & Agriculture Sciences, King Saud University, Riyadh 11451, Saudi Arabia; wahida@ksu.edu.sa; 8 Integrated Molecular Plant Physiology Research, Department of Biology, University of Antwerp, 2020 Antwerpen, Belgium; hamada.abdelgawad@uantwerpen.be

**Keywords:** natural products, phenolic compounds, brine shrimps, protein kinase inhibition, antioxidants

## Abstract

The current study was intended to explore the phytochemical profiling and therapeutic activities of *Putranjiva roxburghii* Wall. Crude extracts of different plant parts were subjected to the determination of antioxidant, antimicrobial, antidiabetic, cytotoxic, and protein kinase inhibitory potential by using solvents of varying polarity ranges. Maximum phenolic content was notified in distilled water extracts of the stem (DW-S) and leaf (DW-L) while the highest flavonoid content was obtained in ethyl acetate leaf (EA-L) extract. HPLC-DAD analysis confirmed the presence of various polyphenols, quantified in the range of 0.02 ± 0.36 to 2.05 ± 0.18 μg/mg extract. Maximum DPPH scavenging activity was expressed by methanolic extract of the stem (MeOH-S). The highest antioxidant capacity and reducing power was shown by MeOH-S and leaf methanolic extract (MeOH-L), respectively. Proficient antibacterial activity was shown by EA-L extract against *Bacillus subtilis* and *Escherichia coli*. Remarkable α-amylase and α-glucosidase inhibition potential was expressed by ethyl acetate fruit (EA-F) and n-Hexane leaf (nH-L) extracts, respectively. In case of brine shrimp lethality assay, 41.67% of the extracts (LC_50_ < 50 µg/mL) were considered as extremely cytotoxic. The test extracts also showed mild antifungal and protein kinase inhibition activities. The present study explores the therapeutic potential of *P*. *roxburghii* and calls for subsequent studies to isolate new bioactive leads through bioactivity-guided isolation.

## 1. Introduction

Plants are nature’s gifts that have been employed for the management of various health-threatening diseases since the early ages [1,2,3]. It has been revealed that approximately 80% of the population across the globe depends upon plant-based therapeutics for healthcare needs [3]. Phytoconstituents are the compounds generated by plants as a defense system against pathogens and predators. They possess certain characteristics that are helpful for the treatment of diseases including antimicrobial, antioxidant, and stimulation as well as inhibition enzymes [4]. Plants contain a wide range of constituents such as alkaloids, tannins, polyphenolics, terpenoids, etc. which are attributable to therapeutic potential [5]. Phytochemicals either isolated compounds or crude extracts, providing opportunity for drug discovery. Human beings seem interested in natural therapeutics as synthetic drugs are associated with adverse effects [6]. Plant-based therapeutic agents are cost-effective and possess fewer side effects as compared to synthetic agents [2].

The current era has noticed the growing enthusiasm of pharmaceutical industries to explore plants for the discovery of new therapeutic moieties [7]. Various commercially available therapeutic agents have been derived from plants such as galegine isolated from *Galega officinalis* L. provided the base for synthesis of metformin which is an antidiabetic drug. Cinchona obtained from *Cinchona officinalis* lead to the development of chloroquine and mefloquine as antimalarial agents [8]. Moreover, vinblastine, vincristine isolated from *Vinca rosea* Linn., and paclitaxel derived from *Taxus brevifolia* are currently available anticancer agents [9,10].

Currently, HPLC is acquiring popularity for the identification of phytochemicals. Qualitative analysis generates a “fingerprint” chromatogram which is helpful for quality control of phytoconstituents. We can also use TLC; however, under certain circumstances it can give false results. HPLC is also helpful for chemosystematics and capable to characterize different species on the basis of their secondary metabolite contents. Reversed-phase HPLC is being widely used for analysis of flavonoids [11].

*Putranjiva roxburghii* Wall. is a moderate size, evergreen tree from the Euphorbiaceae family. It is mainly found in Thailand, Myanmar, Sri Lanka, Bangladesh, Nepal, and Indochina [12,13]. Folklore uses of *P*. *roxburghii* include treatment of rheumatism, muscle twisting, fever, arthralgia, pain, and inflammation [14,15]. Previous studies have revealed the presence of a wide range of triterpenoids including putranjivadione, putranjivanonol, putranjic acid, roxburghonic acid, and roxburgholone, etc. [16,17,18,19]. Despite the traditional use of *P*. *roxburghii* since ancient times for various ailments; scientific evidence regarding the therapeutic potential of this plant and phytochemical profiling is still deficient [16]. Reliability for the therapeutic potential of conventionally used plants requires scientifically valid data. Therefore, the therapeutic effectiveness of plants should be explored to provide a base for the isolation of bioactive leads thus facilitating the drug discovery process [20]. Lack of data on polyphenolic profiling and absence of scientific evidence for the therapeutic potential of *P*. *roxburghii* encouraged us to bridge this research gap. Hence, the present study was carried out to analyze the polyphenols present in different parts of *P*. *roxburghii* and to evaluate their potential implications for reducing oxidative stress. Moreover, the antibacterial, antifungal, α-amylase inhibition, α-glucosidase inhibition, protein-kinase inhibition, and cytotoxic potential of these extracts were also explored.

## 2. Materials and Methods

### 2.1. Acquisition and Identification of Selected Plants

Different plant parts (stems, leaves, and fruits) were acquired from the locality of Quaid-i-Azam University, Islamabad (33.747° N, 73.1356° E) in December 2018. After identification, an authorized sample of the plant was kept in the departmental Herbarium, under voucher number PHM 510 mL.

### 2.2. Reagents and Solvents for Biological Evaluation

PhosPhate buffer was acquired from Riedel-de Haen, Seelze, Germany while Sabouraud dextrose agar from Oxoid, England. Ferric chloride, Potassium ferricyanide, Trichloroacetic acid (TCA), Tryptone soy broth, Surfactin, Doxorubicin, Ascorbic acid, 2,2-diphenyl-1-picrylhydrazyl (DPPH), Nutrient agar, Sea salt, Standard antifungals (clotrimazole and amphotericin B) and Standard antibiotics (cefixime and roxithromycin) were purchased from Sigma Aldrich, Saint Louis, MO, USA. Dried instant yeast was acquired from Fermipan BDH, Poole, England and Medium ISP4 was formulated in laboratory. Brine shrimp “*Artemia salina*” eggs were acquired from Ocean star Int., Coral springs, FL, USA and Tween-20 from Merck-Schuchardt, USA. Microplate reader was purchased from Biotech, Minneapolis, MN, USA, microplate reader Elx 800 while Eppendorf tubes were acquired from Merck, Kenilworth, NJ, USA. Bacterial and fungal strains were acquired from (Microbiologics, Saint Cloud, MN, USA). Eppendorf tubes were taken from Merck, Kenilworth, NJ, USA.

### 2.3. Preparation of Crude Extracts

Plant parts were thoroughly washed and shade-dried for four weeks. After drying, plant parts were crumbled and subjected to the sonication aided maceration in 1000 mL Erlenmeyer flasks for three days. Solvents used for maceration include n-hexane (nH), ethyl acetate (EA), methanol (MeOH), and distilled water (DW), respectively. After the specified duration, filtration was done while marc was macerated in the same solvent for 1 day followed by filtration. All filtrates of the same solvent were merged and subjected to drying by a rotary evaporator (Buchi, Flawil, Switzerland). The crude extracts after complete drying were stored at −80 °C.

Following formula was used to calculate percent extract recovery:

Percent extract recovery (% *w*/*w*) = (x/y) × 100

x = Total weight of the dried extract, y = Total dried weight of the powdered plant material used in extraction, i.e., 500 g each part.

### 2.4. Phytochemical Analysis

Stock solutions of all test extracts were formulated as 4 mg/mL DMSO for phytochemical analysis.

#### 2.4.1. Determination of Total Phenolic Content (TPC)

Initially, 20 µL test extract was shifted to the different wells of 96-well plate with subsequent inclusion of 90 µL Folin–Ciocalteu (FC reagent), 90 µL of sodium carbonate and absorbance was checked after incubation. Gallic acid was taken as positive control and calibration curve was plotted (y = 0.0738x + 0.086) while DMSO was taken as negative control. Results were depicted as the mean of µg gallic acid equivalent (GAE) /mg extract ± SD [21].

#### 2.4.2. Determination of Total Flavonoid Content (TFC)

Initially, 20 µL test extract was shifted to different wells of the 96-well plate with subsequent inclusion of 10 µL (1M) potassium acetate solution, 10 µL (10%) aluminum chloride, and 160 µL distilled water and absorbance was checked after incubation. Quercetin was taken as a positive control and the calibration curve was plotted (y = 0.0535x − 0.0033) while DMSO was employed as negative control. Results were recorded as mean of µg quercetin equivalent (QE)/mg extract ± SD [21].

#### 2.4.3. HPLC-DAD Analysis

High performance liquid chromatography (HPLC) was carried out by Agilent Chem station Rev. B.02-01-SR1 (260), Boulder, Colorado, USA [22]. Two mobile phases were employed including acetonitrile-methanol–water-acetic acid in a ratio of 5:10:85:1 (solvent A) and acetonitrile-methanol-acetic acid in a ratio of 40:60:1 (solvent B). Stock solutions of different polyphenols including cinnamic acid derivatives (caffeic acid, ferulic acid, coumaric acid), benzoic acid derivative (vanillic acid, gallic acid, syringic acid, gentisic acid), flavanol flavonoids (kaempferol, quercetin, myricitin), flavan-3-ol flavonoids (catechin), flavone flavonoid (luteolin, apigenin), naphthoquinone (plumbagin), anthraquinone (emodin), and benzoquinone (thymoquinone). Serial dilutions of stock solutions were prepared, i.e., 10, 20, 50, 100, 200 μg/mL, and a calibration curve was plotted for peak areas at different concentrations of standards. The absorption of samples was checked at 257 nm, 279 nm, 325 nm, and 368 nm and the results were depicted as µg/mg extract. Polyphenols were identified by comparing the retention time of test samples with standards and results were expressed as mean of polyphenolic concentration µg/mg ± S.D.

#### 2.4.4. Reagents and Solvents for Phytochemical Analysis

Ethyl acetate, Methanol, Dimethyl sulfoxide (DMSO), and n-hexane were purchased from Sigma-Aldrich, Schnelldorf, Germany. Folin–Ciocalteu (FC) reagent was acquired from Riedel-de Haen, Germany. Quercetin, Potassium acetate, Gallic acid, and Aluminium chloride were purchased from Sigma Aldrich, Saint Louis, MO, USA. Rotary evaporator was acquired from Buchi, Flawil, Switzerl and while microplate reader was purchased from Biotech USA, microplate reader Elx 800. HPLC was acquired from Agilent Chem station Rev. B.02-01-SR1 (260), Boulder, Colorado, USA. Eppendorf tubes were taken from Merck, Kenilworth, NJ, USA.

### 2.5. Biological Evaluation

#### 2.5.1. Antioxidant Assays

##### DPPH Free Radical Scavenging Assay

At first, each test extract (10 µL) was shifted to the corresponding wells of a 96 well-plate with subsequent inclusion of 190 µL DPPH solution and [23]. The absorbance was checked and % scavenging activity was determined as:

% Scavenging activity = (1 − Abs/Abc) × 100

whereas Abs = Absorbance of sample, Abc = Absorbance of control. Ascorbic acid and DMSO were employed as positive and negative controls, respectively. Results were expressed as the mean of % DPPH scavenging activity ± SD and IC_50_ value was determined for the samples expressing more than 50% scavenging activity.

##### Determination of Total Antioxidant Capacity (TAC)

Initially, each test sample (100 µL) was shifted to Eppendorf tubes with subsequent addition of 900 µL TAC reagent and incubation at 95 °C for 90 min [24]. Ascorbic acid and DMSO were taken as positive control and blank, respectively. Absorbance of the reaction mixture was noted at 630 nm and a calibration curve was plotted (y = 0.0408x − 0.025) while results were illustrated as the mean of µg ascorbic acid equivalent (AAE)/mg extract ± SD.

##### Determination of Total Reducing Power (TRP)

TRP of test extracts was investigated by adopting a previously reported protocol [24]. At first, each test extract (100 µL) was shifted to Eppendorf tubes (Merck, Kenilworth, USA) with the subsequent addition of 200 µL phosphate buffer (0.2 mol/L, pH 6.6) and 250 µL of 1% potassium ferricyanide. Amalgam was incubated and 10% trichloroacetic acid (200 μL) was then added followed by centrifugation. Then, 150 μL aliquots from the supernatant was transferred into the wells of microtiter plate containing FeCl_3_ (50 μL, 0.1%) and absorbance was recorded. Positive and negative controls were ascorbic acid and DMSO. A calibration curve was plotted (y = 0.0754x + 0.1034) while the results were demonstrated as a mean of µg AAE/mg extract ± S.D.

#### 2.5.2. Antimicrobial Assays

##### Antibacterial Assay

The antibacterial activity of test samples was investigated by disc diffusion protocol [25]. Refreshed bacterial cultures [*Pseudomonas aeruginosa* (ATCC15442), *Staphylococcus aureus* (ATCC-6538), *Klebsiella pneumoniae* (ATCC-1705), *Escherichia coli* (ATCC-25922), and *Bacillus subtilis* (ATCC-6633)] (Microbiologics, Saint Cloud, MN, USA) were employed to prepare lawns on nutrient agar plates. Briefly, 5 µL aliquots of every sample were transferred from stock solution (20 mg/mL DMSO) to discs. Subsequently, cefixime and roxithromycin were chosen as positive control while DMSO as a negative control. The discs were implanted on agar plates followed by incubation and zones of inhibition (ZOI) were measured (mm) around each disc. Minimum inhibitory concentration (MIC) was investigated for all the test samples showing ≥12 mm zone of inhibition. For MIC determination, the bacterial inoculum was prepared with a pre-defined density (5 × 10^4^ CFU/mL). Serial dilutions were prepared for each test sample in a 96-well plate by using nutrient broth. Subsequently, 195 μL of inoculum was included and the plate was then incubated. The absorbance of assay plate was recorded after 30 min (zero time reading) and 24 h of incubation and difference was determined. Percent inhibition of bacterial growth was calculated as:

% inhibition = (1 − T_s_/T_c_) × 100

T_s_ is the turbidity of the sample well and T_c_ is the turbidity of the control well. The lowest concentration at which test samples showed ≥90% inhibition of bacterial growth was referred as MIC.

##### Antifungal Assay

Initially, refreshed inoculum was prepared by harvesting the fungal strains, i.e., *Mucor* species (FCBP 0300), *Aspergillus niger* (FCBP 0198), *Aspergillus flavus* (FCBP 0064), *Fusarium solani* (FCBP 0291), and *Aspergillus fumigatus* (FCBP 66) (Microbiologics, Saint Cloud, MN, USA) in Tween 20 solution. Subsequently, 20–25 mL sabouraud dextrose agar (SDA) was taken in petri plates and swabbed with refreshed inoculum (100 µL). The discs loaded with test extracts (5 µL, 20 mg/mL DMSO), clotrimazole (5 µL, 4 mg/mL DMSO) and DMSO (5 µL) were implanted on SDA plates. The petri plates were then incubated and ZOI (mm) around each disc was measured after incubation [26].

#### 2.5.3. Enzyme Inhibition Assays

##### α-Amylase Inhibition Assay

This assay was conducted in accordance with a preceding protocol [24]. First of all, phosphate buffer (15 μL, pH 6.8), α-amylase enzyme (25 µL, 0.0175 U/mL), test sample (10 µL, 4 mg/mL DMSO) and starch solution (40 µL, 2 mg/mL potassium phosphate buffer) were transferred to the corresponding wells of 96 well plate. The plates were kept at 50 °C for 30 min after which HCl (20 µL, 1 M) and iodine reagent (90 µL) were added, and absorbance was checked at 540 nm. An identical protocol was adopted for the formation of negative and positive controls by substituting the test sample with a similar volume of DMSO and acarbose, respectively. Moreover, a blank was prepared by adding an equal quantity of buffer rather than test extract and α-amylase enzyme solution. %enzyme inhibition was calculated as:

%enzyme inhibition = [(Abs − Abn)/(Abb − Abn) × 100]

where, Abs = absorbance of test sample, Abb = absorbance of blank, and Abn = absorbance of negative control.

##### α-Glucosidase Inhibition Assay

This assay was executed by adopting a protocol demonstrated by Nair et al. [27]. Initially, a substrate solution (25 µL, 20 mM), phosphate buffer (69 µL, 50 mM, pH 6.8), test sample (5 µL, 4 mg/mL DMSO), and enzyme solution (1 µL) were transferred to the corresponding wells of 96 well plate. First reading was taken at 405 nm immediately and the amalgam was placed at 37 °C for 30 min. The reaction was quenched by sodium bicarbonate solution (100 µL, 0.5 mM) and the absorbance was again checked. Negative and positive controls were prepared by following a similar protocol just by substituting the test extract with the same amount of DMSO and acarbose, respectively. Following formula was used for the calculation of %enzyme inhibition:

%enzyme inhibition = Ac − As/Ac × 100

Ac = Absorbance of control, As = Absorbance of sample

#### 2.5.4. Preliminary Toxicity Test

##### Brine Shrimp Lethality Assay

This assay was executed by using brine shrimp (*Artemia salina* acquired from Ocean star Int., Coral Springs, FL, USA) larvae in a 96 well plate by following a preceding protocol [22]. *Artemia salina* eggs were allowed to hatch in a dual-chamber punctured tank containing simulated seawater. The chamber filled with eggs was enfolded with aluminum foil while another chamber was kept under a light source. The tank was kept at 30–32 °C for 24–48 h after which nauplii started moving towards the lightened chamber. Subsequently, 10 mature nauplii were taken in each well of a 96-well plate with subsequent inclusion of 150 μL seawater and corresponding volume of test samples to attain final concentrations of 200, 100, and 50 µg/mL. Seawater was added to make the final volume of each well up to 300 μL. Doxorubicin was chosen as positive control while 1% DMSO as negative control. Ultimately, 96 well plate was incubated after which dead nauplii were computed by using an inverted microscope. Following formula was used to calculate %mortality and LC_50_ was calculated by graph pad prism 5 software (GraphPad, San Diego, CA, USA).

% mortality = no. of dead shrimps/total no. of shrimps × 100

##### Protein Kinase Inhibition Assay

Mycelia fragments of *Streptomyces* species were immersed into sterile tryptone soy broth and kept for 24 h. Petri plates containing ISP4 medium were swabbed with refreshed culture (100 μL). Subsequently, discs loaded with a test sample (5 μL, 20 mg/mL DMSO) were implanted on seeded plates. Surfactin and DMSO-loaded discs were employed as positive and negative controls, respectively. The plates were incubated and the diameter of the bald or clear zone (mm) of inhibition was measured as an indicator of protein kinase inhibition potential [26].

### 2.6. Data Analysis

All the assays were conducted in a triplicate manner and the data have been demonstrated as mean ± standard deviation of triplicate analysis. LC_50_ and IC_50_ values were determined through Graph pad prism 5 software (GraphPad, San Diego, CA, USA) while Post Hoc Tukey HSD (Honest significant difference) test was used to analyze the results using the statistical package SPSS (IBM SPSS, Armonk, NY, USA) and *p* < 0.05 (95% confidence interval) was considered as significant. Correlation coefficient (R^2^) of phytochemical activities was calculated by employing the correlation and regression function of Microsoft Excel program (Microsoft, Redmond, WA, USA).

## 3. Results and Discussion

### 3.1. Percent Extract Yield

**Figure 1:**
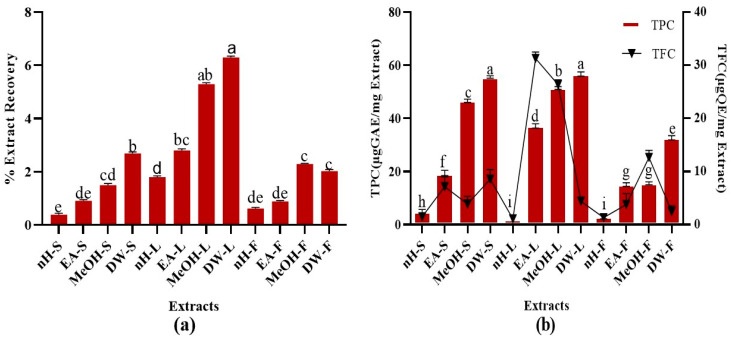
(**a**) Extraction efficiency, (**b**) TPC (Total phenolic content μg GAE/mg) and TFC (Total flavonoid content μg QE/mg) of *P. roxburghii* crude extracts. Assays were conducted in a triplicate manner and the data have been demonstrated as mean ± standard deviation. Significantly different means (*p* < 0.05) are represented by different superscripts (^a^^–^^i^). nH-S; n-Hexane stem, EA-S; ethyl acetate stem, MeOH-S; methanol stem, DW-S; distilled water stem, nH-L; n-Hexane leaf, EA-L; ethyl acetate leaf, MeOH-L; methanol leaf, DW-L; distilled water leaf, nH-F; n-Hexane fruit, EA-F; ethyl acetate fruit, MeOH-F; methanol fruit, DW-F; distilled water fruit.

Percent recovery of test extracts is summarized in Figure 1. The results demonstrated that DW and MeOH extracts of leaf possess maximum extraction efficiency, i.e., 6.3 ± 0.4% and 5.3 ± 0.3%, respectively [28,29]. During the current study, polar solvents showed maximum extraction efficiency. Water-soluble phytoconstituents mainly present in plants include flavonoids, tannins, terpenoids, quinones, etc. [30,31]. The presence of some of these metabolites might be attributable to the maximum extraction efficiency of DW and MeOH leaf extracts. The results were in conformity with a previous study in which non-polar extracts of *Ajuga bracteosa* Wall. showed less extraction efficiency as compared to polar ones [25].

### 3.2. Phytochemical Analysis

#### 3.2.1. Total Phenolic Content (TPC)

A remarkable TPC was noted in test extracts. Maximum TPC was notified in DW extracts of stem and leaf, i.e., 55.23 ± 0.10 µg GAE/mg extract while least TPC was found in nH extracts of leaf and fruit, i.e., 2.06 ± 0.03 µg GAE/mg extract, respectively (Figure 1). TPC notified in our research was somewhat higher than a recently published study which revealed that *P. roxburghii* leaf (hydroethanol, 30:70) extract contains 46.58 ± 2.52 mg/g GAE polyphenolic content [32]. Alterations in agro-climatic conditions accompanied with temperature and rainfall impart a significant impact on the amount of phytoconstituents within similar species of plants growing in different regions [33]. Hence, differences in solvent composition and plant collection sites might be responsible for the variations of estimated TPC in both studies. Current study investigated the phenolic content in fruit and stem parts of *P. roxburghii* as well and employed a wide range of solvents [32]. Phenolics are secondary metabolites of plants which help to counteract different pathogenic microbes and impart colors to the plants. They are widely distributed in all plant parts and hence, act as an essential component of the human diet [34]. Phenolics are highly effective to counteract oxidative stress-related ailments such as neurodegenerative diseases, cancer, diabetes, ageing, and cardiovascular diseases [35]. Phenolic compounds perform antioxidant activity by the virtue of hydrogen donating action and chelation of metal ions involved in the generation of free radicals [36,37]. The presence of hydrophobic benzenoid rings enables the phenolic constituents to interact with proteins and to inhibit enzymes involved in free radical production [36,38]. These compounds can also act as neuroprotective, gastroprotective, antipyreticm, antiatherosclerotic, cardioprotective, antiulcer [39], anti-inflammatory [35], antibacterial [40], antifungal [41], antiviral [42], and antitumor agents [35,43], antidiabetic, anxiolytic [39]. Hence, the presence of a remarkable quantity of phenolic content in *P. roxburghii* might be accountable to its promising therapeutic potential.

#### 3.2.2. Total Flavonoid Content (TFC)

A significant TFC was observed in crude extracts. Highest TFC was found in EA and MeOH extracts of leaf, i.e., 30.62 ± 0.31, 27.25 ± 0.05 µg QE/mg extract, respectively (Figure 1). Minimum TFC was notified in nH extracts of stem, leaf, and fruit, i.e., 1.04 ± 0.02 µg QE/mg extract. The results were in close resemblance to a previous study which revealed that the hydroethanolic extract of *P. roxburghii* leaf part contains 29.0 ± 2 mg/g QE flavonoid content [32]. It was found that EA leaf extract contains a greater quantity of flavonoids than MeOH extract. Flavonoids are an important class of polyphenolics containing benzo-γ-pyrone moiety and possess a wide range of therapeutic activities [44]. Flavonoids contain a flavan core, consisting of 15 carbon atoms organized in three rings. Depending upon the oxidation state of the central core, flavonoids are categorized as: anthocyanins, flavanones, isoflavones, flavones, flavanols, and flavonols. The structural differences in each subclass are due to the extent and pattern of prenylation, methoxylation, glycosylation, and hydroxylation [34]. Flavonoids can act as antimicrobial, anti-inflammatory, immunomodulatory, enzyme inhibitor, antiviral, antiparasitic, cardioprotective, cytotoxic, anti-tumor, antiaging agents [45,46]. A wide range of flavonoids have been isolated from *roxburghii* species such as catechin and quercetin in *Rosa roxburghii* Tratt. [47], isorhamnetin, rhamnazin, roxburoside from *Anoectochilus roxburghii* Wall. [48], and putraflavone from *P. roxburghii* [49]. During the present study, the flavonoid presence in polar extracts of *P. roxburghii* referred it to be a potential reservoir of antioxidants.

#### 3.2.3. HPLC-DAD Analysis of Polyphenols

**Figure 2:**
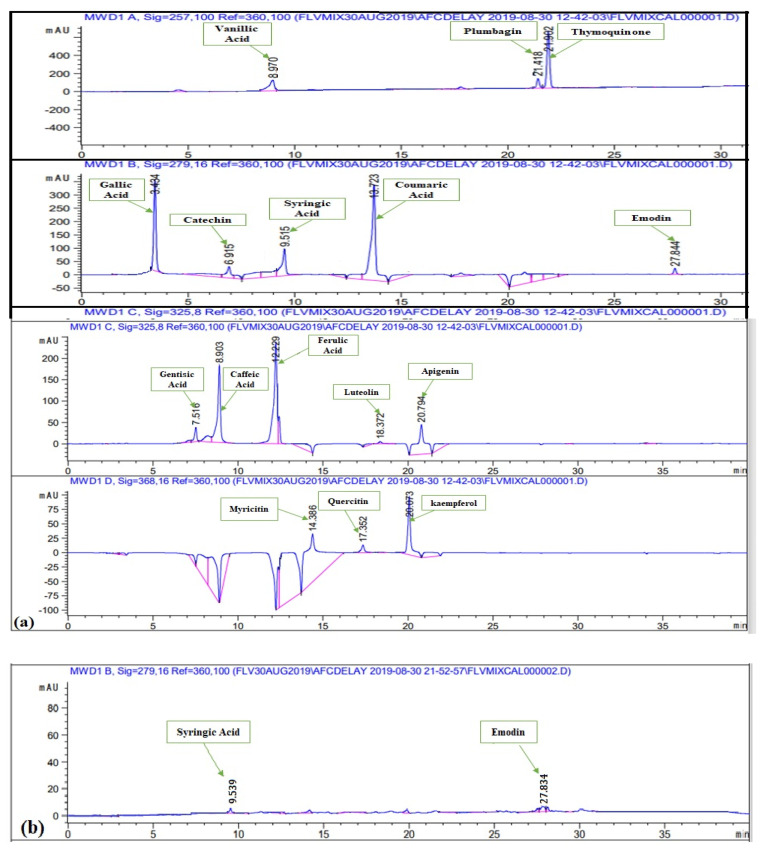

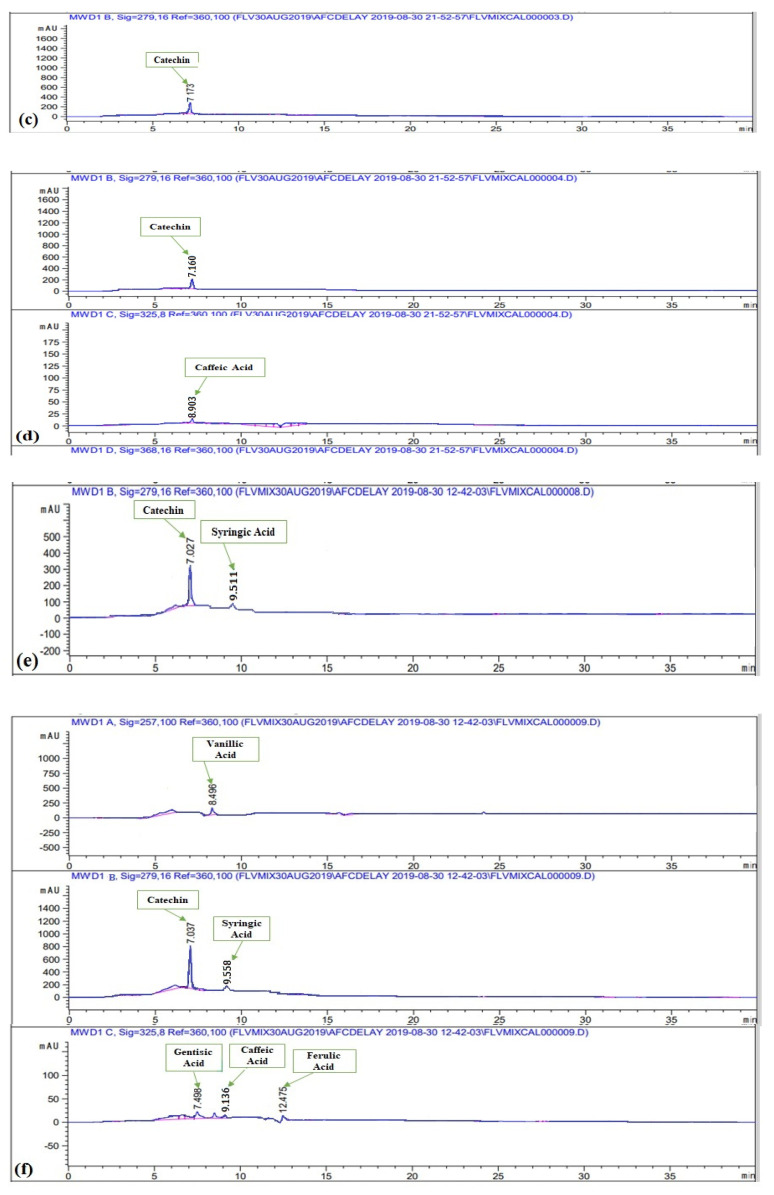

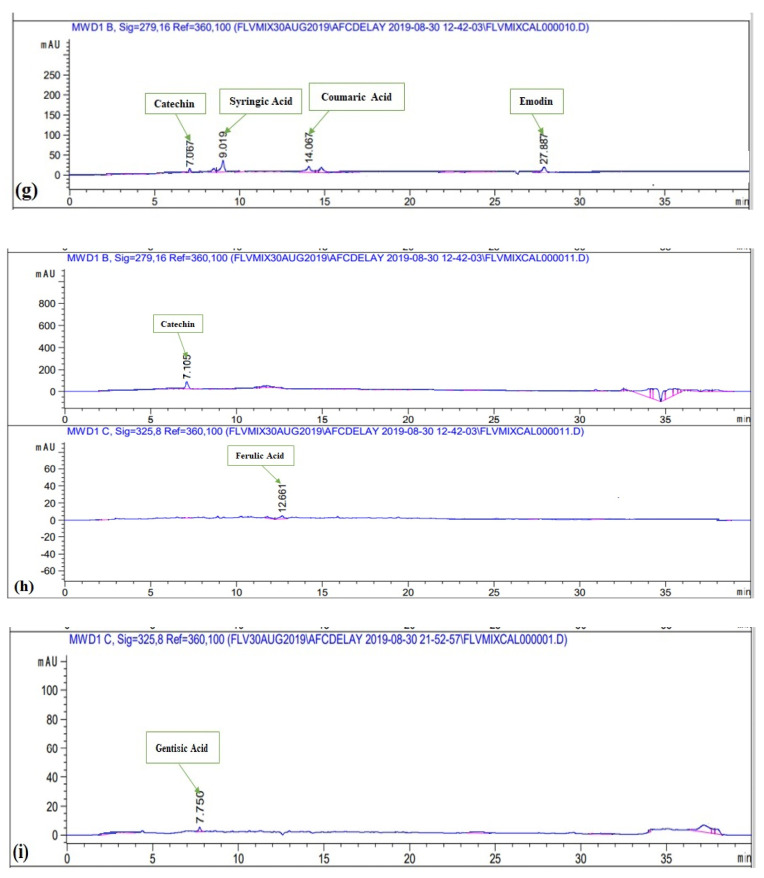
HPLC chromatograms of (**a**) standard polyphenols (**b**) EA-S (**c**) MeOH-S (**d**) DW-S (**e**) MeOH-L (**f**) DW-L (**g**) EA-F (**h**) MeOH-F (**i**) DW-F.

Polyphenols were quantified in various extracts of *P. roxburghii* by HPLC-DAD analysis (using 16 references) (Figure 2). Caffeic Acid, ferulic acid, coumaric acid, vanillic acid, gallic acid, syringic acid, gentisic acid, catechin, and emodin were present in significant amounts (Table 1). Whereas kaempferol, quercetin, myricitin, luteolin, apigenin, plumbagin, and thymoquinone were not present in any crude extract. Maximum number of polyphenols was detected in DW-L extract including caffeic acid (0.35 ± 0.06 μg/mg extract), ferulic acid (1.02 ± 0.51 μg/mg extract), vanillic acid (0.26 ± 0.32 μg/mg extract), syringic acid (0.92 ± 0.52 μg/mg extract), gentisic acid (1.19 ± 0.09 μg/mg extract), and catechin (2.05 ± 0.18 μg/mg extract). It was followed by EA-F extract which contains coumaric acid (0.02 ± 0.36 μg/mg extract), syringic acid (0.76 ± 0.42 μg/mg extract), catechin (0.56 ± 0.56 μg/mg extract), and emodin (0.87 ± 23 μg/mg extract). Subsequently, MeOH-F extract contains ferulic acid (0.44 ± 0.26 μg/mg extract) and catechin (0.61 ± 0.67 μg/mg extract), DW-S extract contains caffeic acid (0.26 ± 0.03 μg/mg extract) and catechin (0.69 ± 0.18 μg/mg extract) while EA-S extract contains syringic acid (0.17 ± 0.04 μg/mg extract) and emodin (0.07 ± 0.16 μg/mg extract). Moreover, MeOH-S extract contains catechin (1.02 ± 0.52 μg/mg extract) while DW-F extract contains gentisic acid (0.49 ± 0.82 μg/mg extract). The detection of these polyphenols derives a direct correlation of plants potential with notified biological activities. All reported polyphenols possess a wide range of therapeutic activities. Caffeic acid has antioxidant [50], anticancer, anti-fibrosis, antihypertension [51,52], anti-hepatitis B virus [53] anti-inflammatory, anti-coagulant activities [54]. Ferulic acid has anti-oxidant [55], cytotoxic [56], antiallergic, antiviral, anti-inflammatory, antimicrobial, anticoagulant, and hepatoprotective properties [57,58]. Reported activities of coumaric acid include antioxidant [59], anti-microbial [60], anti-leishmaniasis, cytotoxic [61]. Vanillic acid has hepato-protective [62] and anti-oxidant activities [63] while syringic acid has hepato-protective, neuro-protective, cardio-protective, anti-inflammatory, and hypoglycemic potential [64]. Gentisic acid possesses fibro growth factor inhibition [65], antimicrobial, antioxidant, anti-inflammatory, hepatoprotective, and neuroprotective activities [66]. Reported activities of catechin are anti-cancer [67], anti-microbial [68], hypolipidemic [69], vasodilator, antispasmodic, and bronchodilator activities [70]. Finally, emodin has anti-inflammatory, anti-cancer, antimicrobial, hepatoprotective potential [71,72]. Hence, detection of a wide range of polyphenols further manifests the therapeutic potential of *P. roxburghii*. Best to our knowledge, HPLC-DAD analysis of *P. roxburghii* was reported for the first time in the present study. Previously, HPLC analysis of polyphenols was carried out for *Rosa roxburghii* Tratt. leaf extracts. Approximately, 30 polyphenols were detected, which mainly includes gallic acid, myricetin, (+)-catechin, quercetin-3-O-galactoside, arbutin, and 3-hydroxybenzoic acid [73]. HPLC analysis of *Pinus roxburghii* barks and needles was carried out and quercetin was identified as the most abundant flavonol [11].

### 3.3. Biological Evaluation

#### 3.3.1. Antioxidant Assays

##### DPPH Assay

**Figure 3:**
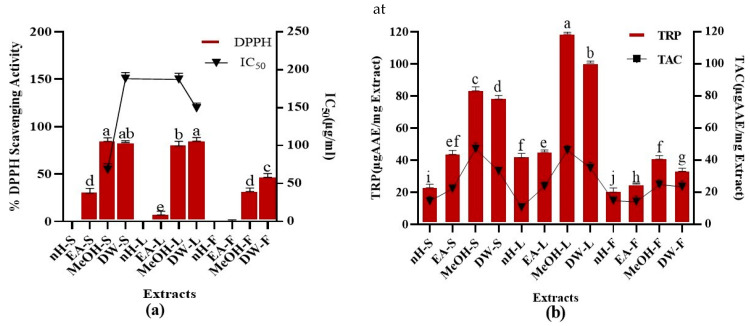
Demonstration of (**a**) %DPPH radical scavenging activity (at 40 µg/mL concentration of test extracts) (**b**) TAC and TRP (µg AAE/mg) of *P. roxburghii* crude extracts. Assays were conducted in a triplicate manner and the data have been demonstrated as mean ± standard deviation. Significantly different means (*p* < 0.05) are represented by different superscripts (^a–j^).

Metabolic processes within the body and environmental factors produce free radicals. Free radicals mainly include reactive oxygen species (ROS) which cause various ailments including ageing, carcinogenesis, mutagenesis, and cardiovascular abnormalities. Antioxidants are the agents, which counteract the effects of free radicals and limit oxidative stress [74]. DPPH assay is a standard method to investigate the free radical scavenging ability of test samples [75]. The percent free radical scavenging activity (%FRSA) of test extracts was analyzed and results are given in Figure 3. Maximum %FRSA was shown by MeOH-S and DW-L extracts (86 ± 0.56%) with IC_50_ values of 68 ± 0.43, 149 ± 0.21 µg/mL, respectively. Whereas, nH extracts of stem, leaf, and fruit were unable to express any free radical scavenging activity. Moreover, ascorbic acid showed 78% FRSA with IC_50_ of 14.56 µg/mL. We calculated the correlation of %FRSA with TPC (R^2^ = 0.8084) and TFC (R^2^ = 0.0211) which demonstrates that phenolic compounds other than flavonoids are mainly attributable to free radical scavenging activity. Previously reported %FRSA of methanolic stem extracts of *P. roxburghii* was somewhat lower than calculated during our commenced research. Moreover, the current study revealed that DW-L extract of *P. roxburghii* also possesses a promising free radical scavenging potential. Highest extraction efficiency of DW-L extract provides another perspective to employ this extract as an antioxidant. Overall, our results were in accordance with a previous study which revealed that polar extracts possess better scavenging potential than non-polar extracts [25].

##### Total Antioxidant Capacity (TAC)

TAC of test extracts was calculated as shown in Figure 3. Maximum TAC was shown by MeOH-S and MeOH-L extracts, i.e., 48.65 ± 0.52, 46.07 ± 0.07 μg AAE/mg extract, respectively. Minimum TAC (10.91 ± 0.05 μg AAE/mg extract) was expressed by nH-L extract. We noticed a remarkable relationship between TAC and TPC (R^2^ = 0.744) which demonstrates that phytoconstituents producing antioxidant activity mainly belong to polyphenols. Moreover, the correlation between TAC and TFC (R^2^ = 0.1419) shows that flavonoids are not the main contributor to the antioxidant activity of test extracts. During a previous study, the estimated TAC of *P. roxburghii* stem part was significantly lower than that of the current study. This might be due to the fact that varying agro-climatic conditions can impact the type and quantity of phytoconstituents [41]. Moreover, the antioxidant capacity of different parts of *P. roxburghii* except stems, has been investigated for the first time [9]. Plant-derived antioxidants are considered better than synthetic antioxidants as they possess better compatibility with the human body [41], hence provide a justification for the exploitation of herbal products for therapeutic appraisal.

##### Total Reducing Power (TRP)

TRP was investigated for crude extracts and results are depicted in Figure 3. Maximum TRP was expressed by MeOH-L and DW-L extracts, i.e., 118.59 ± 0.08, 101.28 ± 0,09 µg AAE/mg extract while nH-F extract showed the least TRP, i.e., 20.48 ± 0.52 µg AAE/mg extract, respectively. A positive relation was notified between TPC and TRP (R^2^= 0.7251) in contrast to the correlation between TFC and TRP (R^2^ = 0.1495) which demonstrates that phenolic constituents other than flavonoids are mainly accountable for reducing potential of plants. Reducing power of *P. roxburghii* stem part was previously investigated; however, the current study revealed that leaf part of this plant possesses better antioxidant potential [12]. Reducing power is mainly attributable to the presence of reductones which cleaves the free radical chain by hydrogen atom donation and participates in antioxidant action. Multiple studies have notified a close relationship between antioxidant activity and reducing power of crude extracts which is in support of our current research results [24,76].

#### 3.3.2. Antimicrobial Assays

##### Antibacterial Assay

Antibacterial property of test extracts (100 µg per disc) was investigated against gram-negative (*K*. *pneumoniae*, *P. aeruginosa*, *E*. *coli*) and gram-positive (*B*. *subtilis*, *S*. *aureus*) bacteria as shown in Table 2. Test extracts exhibiting zone of inhibition ≥ 12 mm were further subjected for MIC evaluation by employing the broth microdilution method. EA-L extract was highly active against *B*. *subtilis* (MIC 3.7 µg/mL), *K*. *pneumoniae* (MIC 33.3 µg/mL), and *E*. *coli* (MIC 3.7 µg/mL) with 24 ± 0.5, 20 ± 0.50, 23 ± 0.76 mm ZOIs, respectively. The ZOIs generated by the test extracts were equivalent to those produced by standards, i.e., roxithromycin and cefixime. Moreover, DMSO taken as negative control was unable to show inhibition. Overall, polar extracts showed better antibacterial potential than non-polar extracts. In a previous study, antibacterial potential of *P. roxburghii* MeOH leaf part was investigated, and results were in somewhat resemblance to our study. However, EA leaf extract was investigated for the first time, and it was noted that this extract possessed stronger antibacterial activity than previously reported MeOH leaf extract. Phenolics impart toxic effects to microbes either through interaction with sulfhydryl groups or proteins resulting in enzyme inhibition. Moreover, polyphenols generate heavy complexes with proteins which interact with bacterial adherence and disrupt the receptors on cell surface [26,77]. During the current study, a proficient amount of TPC and TFC was notified which might contribute to the antibacterial activity.

##### Antifungal Assay

During present study, MeOH-F extract (100 µg/disc) showed slight activity against *A*. *flavus* and *A*. *fumigatus* with 7 ± 0.89 and 8 ± 0.98 mm ZOIs, respectively and MIC value of >100 µg/mL. MeOH-S extract was moderately active against *A*. *flavus* with 12 ± 0.98 ZOI and >100 µg/mL MIC while other extracts were unable to show antifungal activity. Clotrimazole (10 µg/disc) showed 20 ± 0.57 to 31 ± 1.1 mm ZOIs. A previous study reported the promising antifungal activity of seed and pericarp of *P. roxburghii*. However, during the present study; stem, leaf, and fruit parts of *P. roxburghii* showed slight to moderate antifungal active. Moreover, fungal strains used in current study were different from those used in previous study. Flavonoids and tannins can form complexes with extracellular proteins present in the cell wall of fungi and rupture the fungal membrane [78,79]. *P. roxburghii* contains tannins (ellagic acid, gallic acid, gallocatechin, ellagi- and gallo-tannins) and flavonoids (bioflavones) which might be a contributor to antifungal activity [49].

#### 3.3.3. Enzyme Inhibition Assays

##### α-Amylase Inhibition Assay

**Figure 4:**
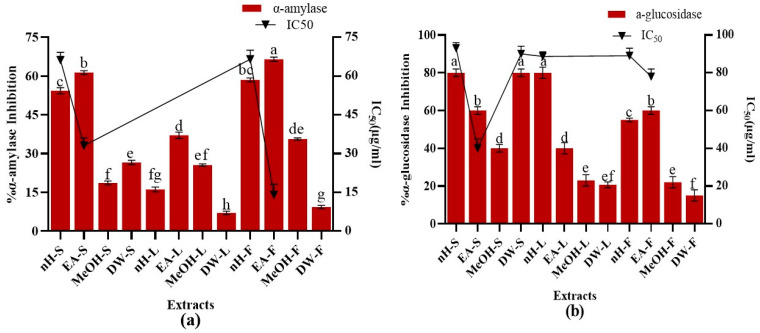
Demonstration of (**a**) α-amylase inhibition and (**b**) α-glucosidase inhibition potential of *P. roxburghii* crude extracts. Assays were conducted in a triplicate manner and the data have been demonstrated as mean ± standard deviation. Significantly different means (*p* < 0.05) are represented by different superscripts (^a–h^).

The suppression of carbohydrate cleaving enzymes, i.e., α-amylase and α-glucosidase is a promising approach to reduce the blood glucose concentration in case of diabetes [27]. Inhibition of both enzymes impedes carbohydrate digestion and enhance the time required for carbohydrate digestion, limits rate of glucose absorption and ultimately prevents post-prandial rise in plasma glucose level [80]. During the present study, α-amylase inhibition potential of crude extracts was investigated and results are depicted in Figure 4. Maximum α-amylase inhibition activity was expressed by EA-F and EA-S extracts, i.e., 67.37 ± 0.05%, 61.37 ± 0.06% with IC_50_ values, i.e., 14.28 ± 0.9, 33.26 ± 0.5µg/mL, respectively. Acarbose was employed as standard which showed 80.45 ± 0.76% α-amylase inhibition with IC_50_ value of 34.85 ± 0.21 μg/mL. Results were in accordance with previous studies which reported that less polar extracts exhibited improved enzyme inhibition activity than highly polar extracts. These studies also notified a direct relationship between the concentration of phenolics and flavonoids with α-amylase inhibition activity [81,82]. However, the current study did not notice any significant relation of TPC (R^2^ = 0.343), and TFC (R^2^ = 0.0008) with α-amylase inhibition potential.

##### α-Glucosidase Inhibition Assay

Antidiabetic activity of *P. roxburgii* was further confirmed by estimation of α-glucosidase inhibition potential of crude extracts and results are depicted in Figure 4. Maximum α-glucosidase inhibition (80 ± 0.78%) was shown by nH-L, nH-S, and DW-S extracts with IC_50_ values, i.e., 90.21 ± 0.02, 93 ± 0.078, 94.11 ± 0.99 µg/mL, respectively. Just like α-amylase inhibition, there was no positive relationship of TPC (R^2^ = 0.1971) and TFC (R^2^ = 0.1231) with α-glucosidase inhibition. Acarbose and miglitol are commercially available α-glucosidase inhibitors which hinder the carbohydrate absorption and reduce post-prandial escalation in glucose levels [83]. However, these agents possess severe gastrointestinal adverse effects such as diarrhea and flatulence [84]. α-amylase inhibitors have been reported to be present in plants which helps them to combat predators [85].

A detailed study was executed for the first time to investigate α-amylase and α-glucosidase inhibition potential of different parts of *P. roxburghii*. Previous study demonstrated that plants containing tannins such as *Camelia sinensis* *Var*., *Artocarpus heterophyllus* Lam., *Persea Americana* Mil., and *Syzygium polyanthum* Walp. are potent inhibitors of α-glucosidase [86]. A wide range of tannins such as ellagic acid, gallic acid, gallocatechin, ellagi- and gallo-tannins are present in *P. roxburghii* might be involved behind the α-glucosidase inhibition activity [49]. However, further studies are needed to prove this hypothesis.

#### 3.3.4. Preliminary Toxicity Potential

##### Brine Shrimp Lethality Assay

Brine shrimp lethality assay is a preliminary method to investigate the cytotoxic effect of test samples [87]. Brine shrimp lethality potential was noticed to be in direct relation to the concentration of test extracts (Table 3). Out of the twelve extracts subjected for estimation of cytotoxic activity, 41.67% of the extracts (LC_50_ < 50 µg/mL) were considered as extremely cytotoxic while 33.33% of crude extracts (LC_50_ ≥ 50 but ≤ 200 μg/mL) were classified as moderately cytotoxic. Other 25% of the extracts (>200 μg/mL) were categorized as weakly cytotoxic. Overall, DW-F extract was noticed as most cytotoxic extract showing LC_50_ 9.36 ± 0.91 µg/mL while LC_50_ of doxorubicin (positive control) was 5.93 µg/mL. Previously, brine shrimp lethality potential of methanolic seed extract was investigated and mild cytotoxicity potential was reported [88]. Undertaken study was carried out on various other parts of *P. roxburghii* and promising brine shrimp lethality potential of nH and DW extracts of fruit was revealed for the first time. During the present study, 100% of the test extracts exhibited LC_50_ values of less than 1000 μg/mL, referring to the existence of cytotoxic phytometabolites in the subject plant.

##### Protein Kinase Inhibition Assay

Protein kinase regulates multiple functions of the cell cycle including apoptosis, metabolism, growth, and differentiation. In the case of human cancers, irregularities of growth factor signaling pathways were notified. Hence, protein kinase inhibition can serve as a target for drug development by overcoming the irregularities in signaling pathways [89]. Protein kinase inhibition activity of the test extracts (100 µg/disc) was investigated as well (Table 3). DW and nH fruit extracts showed 8 ± 0.4 and 7 ± 0.97 mm bald phenotype zones, respectively while no clear zone was found. The remaining extracts were unable to express any protein kinase inhibition activity. The absence of ZOI in case of DMSO confirmed the non-toxic effect of negative control. Protein kinase is involved in the regulation of various processes of cell cycle, i.e., control of cycle growth, metabolism, differentiation, and apoptosis. Abnormalities of growth factor signaling pathways have been notified in various forms of human cancer. Hence, protein kinase inhibitors capable to repair the dysregulations in these signaling pathways can act as promising targets for anticancer drug development [89]. Previously, this plant has shown significant anticancer potential against different cancer cell lines. A combined herbal therapy comprised of *Punica granatum* and *P. roxburghii* showed strong cytotoxic activity against HepG2 hepatocellular carcinoma cell line in correlation with its antioxidant effect, hence proving the significant anticancer potential of the subject plant in the current study [90]. Not only the organic solvents extracts but the green silver nanoparticles synthesized using *P. roxburghii* leaves extracts depicted stupendous inhibitory activity against PANC-1, HCT-116 and MDA-MB-231 cell lines showing its potential to reduce the proliferation of cancerous cells [91]. During the present study, *P. roxburghii* DW extract showed mild protein kinase inhibition activity. The activity is supported by the results of a previous finding in which water extracts of *Sargassum oligocystum* depicted significant cytotoxic and antiproliferative activity against K562 and Daudi cell lines [92]. The linkage of protein kinase inhibitory activity and anticancer effects persuaded to draw the relation between previous findings and the results of the current studies. Although, a detailed study has been conducted for the first time to investigate the protein kinase inhibition potential of multiple parts of *P. roxburghii*.

## 4. Conclusions

Undertaken study demonstrates the polyphenolic analysis and pharmacological potential of crude extracts. Overall, leaf part showed maximum biological activities as compared to the stem and fruit parts of *P. roxburghii*. In case of leaf part DW and MeOH extracts were highly active whereas EA and nH extracts also showed some biological activities. The present study suggests that *P. roxburghii* crude extracts are a potential reservoir of phytoconstituents instigating the considerable antioxidant, antibacterial, antidiabetic, and cytotoxic compounds. These bioactive phytoconstituents could act as unique frameworks in a quest for innovative drugs. we recommend that subsequent studies should be carried out for the isolation of bioactive compounds from *P. roxburghii* (especially DW-L extract), which can be considered as potential candidates for the treatment of different ailments.

## Figures and Tables

**Table 1:** Chemical profiling (μg/mg extract) of stem, leaf and fruit parts of *P. roxburghii* using HPLC-DAD.

Class	Polyphenols	EA-S	MeOH-S	DW-S	EA-L	MeOH-L	DW-L	EA-F	MeOH-F	DW-F
Cinnamic acid derivatives	Caffeic Acid	--	--	0.26 ± 0.03 ^m^	--	--	0.35 ± 0.06 ^l^	--	--	--
	Ferulic acid	--	--	--	--	--	1.02 ± 0.51 ^c^	--	0.44 ± 0.26 ^k^	--
	Coumaric Acid	--	--	--	--	--		0.02 ± 0.36 ^p^	--	--
Benzoic acid derivative	Vanillic Acid	--	--	--	--	--	0.26 ± 0.32 ^m^	--	--	--
	Gallic Acid	--	--	--	--	--	--	--	--	--
	Syringic Acid	0.17 ± 0.04 ^n^	--	--	--	0.44 ± 0.21 ^j^	0.92 ± 0.52 ^d^	0.76 ± 0.42 ^f^	--	--
	Gentisic Acid	--	--	--	--	--	1.19 ± 0.09 ^b^	--	--	0.49 ± 0.82 ^j^
Flavonol flavonoids	Kaempferol	--	--	--	--	--	--	--	--	--
	Quercetin	--	--	--	--	--	--	--	--	--
	Myricitin	--	--	--	--	--	--	--	--	--
Flavan-3-ol flavonoids	Catechin	--	1.02 ± 0.52 ^c^	0.69 ± 0.18 ^g^	--	0.74 ± 0.01 ^fg^	2.05 ± 0.18 ^a^	0.56 ± 0.56 ^i^	0.61 ± 0.67 ^h^	--
Flavone flavonoid	Luteolin	--	--	--	--	--	--	--	--	--
	Apigenin	--	--	--	--	--	--	--	--	--
*Naphthoquinone*	Plumbagin	--	--	--	--	--	--	--	--	--
Anthraquinone	Emodin	0.07 ± 0.16 ^o^	--	--	--	--	--	0.87 ± 23 ^e^	--	--
*Benzoquinone*	Thymoquinone	--	--	--	--	--	--	--	--	--

-- = not detected. HPLC analysis was carried out in a triplicate manner and the data have been demonstrated as mean ± standard deviation. The values with different superscripts (^a–p^) demonstrate significantly (*p* < 0.05) different mean values.

**Table 2:** Antibacterial activity and MIC values of *P. roxburghii* test extracts.

Extract Codes	Zone of Inhibition (mm) at 100 μg/disc and MIC (µg/mL)									
	S. A	MIC	B. S	MIC	P. A	MIC	K. P	MIC	E. C	MIC
nH-S	7 ± 0.29 ^de^	--	11 ± 0.2 ^de^	--	7 ± 0.15 ^bc^	--	6 ± 0.87 ^ef^	--	11 ± 0.51 ^e^	--
EA-S	7 ± 0.31 ^de^	--	9 ± 0.5 ^e^	--	8 ± 0.31 ^b^	--	7 ± 0.35 ^e^	--	6 ± 0.50 ^g^	--
MeOH-S	10 ± 0.36 ^c^	--	12 ± 0.7 ^d^	100 ^a^	7 ± 0.10 ^bc^	--	10 ± 0.76 ^d^	--	13 ± 0.50 ^d^	100 ^a^
DW-S	7 ± 0.36 ^de^	--	3 ± 0.5 ^g^	--	6 ± 0.10 ^c^	--	12 ± 0.17 ^c^	100 ^a^	20 ± 0.50 ^b^	33.3 ^b^
nH-L	8 ± 0.7 ^d^	--	12 ± 0.5 ^d^	100 ^a^	6 ± 0.31 ^c^	--	7 ± 0.55 ^e^	--	7 ± 0.31 ^fg^	--
EA-L	6 ± 0.3 ^e^	--	24 ± 0.5 ^a^	3.7 ^c^	7 ± 0.15 ^bc^	--	20 ± 0.50 ^a^	33.3 ^b^	23 ± 0.76 ^a^	3.7 ^c^
MeOH-L	8 ± 0.36 ^d^	--	20 ± 0.45 ^b^	33.3 ^b^	6 ± 0.21 ^c^	--	14 ± 0.76 ^bc^	100 ^a^	12 ± 0.31 ^de^	100 ^a^
DW-L	9 ± 0.31 ^cd^	--	9 ± 0.5 ^e^	--	7 ± 0.25 ^bc^	--	12 ± 0.51 ^c^	100 ^a^	6 ± 0.76 ^g^	--
nH-F	5 ± 0.15 ^f^	--	7 ± 0.7 ^f^	--	7 ± 0.15 ^bc^	--	7 ± 0.50 ^e^	--	10 ± 0.15 ^ef^	--
EA-F	13 ± 0.12 ^b^	100 ^a^	8 ± 0.5 ^ef^	--	6 ± 0.15 ^c^	--	19 ± 0.58 ^ab^	33.3 ^b^	8 ± 0.58 ^f^	--
MeOH-F	7 ± 0.31 ^de^	--	12 ± 0.2 ^d^	100 ^a^	5 ± 0.25 ^cd^	--	7 ± 0.31 ^e^	--	18 ± 0.5 ^c^	--
DW-F	10 ± 0.32 ^c^	--	7 ± 0.3 ^f^	--	5 ± 0.33 ^cd^	--	18 ± 0.29 ^b^	33.3 ^b^	7 ± 0.50 ^fg^	--
Rox	23 ± 0.54 ^a^	1.11 ^b^	17 ± 0.3 ^c^	3.33 ^c^	--	--	--	--	--	--
Cefix	--	--	--	--	22 ± 0.89 ^a^	1.11	20 ± 1.2 ^a^	1.11 ^c^	20 ± 1.5 ^b^	3.33 ^c^
DMSO	--	--	--	--	--	--	--	--	--	--

-- = no activity. S. A = *Staphylococcus aureus*, B. S = *Bacillus subtilis*, P. A = *Pseudomonas aeruginosa*, K. P = *Klebsiella pneumoniae*, E. C = *Escherichia coli*. Rox = Roxithromycin, Cefix = Cefixime. Assay was conducted in a triplicate manner and the data have been demonstrated as mean ± standard deviation. The values with different superscripts (^a–g^) depict significantly (*p* < 0.05) different mean values.

**Table 3:** Brine shrimp lethality and protein kinase inhibition potential of crude extracts of *P. roxburghii*.

Extract Codes	% Brine Shrimp Mortality					Protein Kinase Inhibition	
	200 (μg/mL)	100 (μg/mL)	50 (μg/mL)	25 (μg/mL)	LC_50_ (µg/mL)	Clear Zone (mm)	Bald Zone (mm)
nH-S	70 ± 10 ^b^	40 ± 7.5 ^d^	2 ± 11.5 ^g^	20 ± 0 ^e^	130.93 ± 0.56 ^b^	--	--
EA-S	100 ± 0 ^a^	100 ± 0 ^a^	50 ± 5.7 ^b^	30 ± 0 ^d^	39.52 ± 0.42 ^f^	--	--
MeOH-S	100 ± 0 ^a^	40 ± 5.7 ^d^	40 ± 0 ^c^	30 ± 7.5 ^d^	88.51 ± 0.59 ^e^	--	--
DW-S	100 ± 0 ^a^	50 ± 0 ^c^	0 ± 0 ^h^	0 ± 0 ^g^	100 ± 0.67 ^c^	--	--
nH-L	30 ± 0 ^c^	30 ± 0 ^e^	0 ± 0 ^h^	0 ± 0 ^g^	>200 ± 0.73 ^a^	--	--
EA-L	100 ± 5.7 ^a^	100 ± 0 ^a^	70 ± 0 ^a^	60 ± 7.5 ^b^	20 ± 1.16 ^g^	--	--
MeOH-L	30 ± 11.5 ^c^	20 ± 0 ^f^	10 ± 0 ^f^	10 ± 5.7	>200 ± 1.52 ^a^	--	--
DW-L	100 ± 0 ^a^	50 ± 0 ^c^	30 ± 5.7 ^d^	20 ± 0 ^e^	93.1 ± 0.36 ^d^	--	--
nH-F	100 ± 0 ^a^	100 ± 0 ^a^	70 ± 0 ^a^	70 ± 0 ^a^	18.84 ± 0.49 ^gh^	--	7 ± 0.97 ^ab^
EA-F	100 ± 7.5 ^a^	90 ± 7.5 ^b^	50 ± 0 ^b^	40 ± 0 ^c^	35.5 ± 0.53 ^g^	--	--
MeOH-F	30 ± 5.7 ^c^	16 ± 0 ^g^	16 ± 0 ^e^	10 ± 0 ^f^	>200 ± 1.21 ^a^	--	--
DW-F	100 ± 0 ^a^	100 ± 0 ^a^	0 ± 0 ^h^	60 ± 0 ^b^	9.36 ± 0.91 ^i^	--	8 ± 0.4 ^a^

-- = not detected. Assays were conducted in a triplicate manner and the data have been demonstrated as mean ± standard deviation. The values with different superscripts (^a–i^) depict significantly (*p* < 0.05) different mean values.

## Data Availability

Data sharing not applicable.

## Sample Availability

The crude extracts prepared for the current study are available from the authors.
